# Genotype-Dependent Efficacy of a Dual PI3K/mTOR Inhibitor, NVP-BEZ235, and an mTOR Inhibitor, RAD001, in Endometrial Carcinomas

**DOI:** 10.1371/journal.pone.0037431

**Published:** 2012-05-25

**Authors:** Keiko Shoji, Katsutoshi Oda, Tomoko Kashiyama, Yuji Ikeda, Shunsuke Nakagawa, Kenbun Sone, Yuichiro Miyamoto, Haruko Hiraike, Michihiro Tanikawa, Aki Miyasaka, Takahiro Koso, Yoko Matsumoto, Osamu Wada-Hiraike, Kei Kawana, Hiroyuki Kuramoto, Frank McCormick, Hiroyuki Aburatani, Tetsu Yano, Shiro Kozuma, Yuji Taketani

**Affiliations:** 1 Department of Obstetrics and Gynecology, Faculty of Medicine, The University of Tokyo, Tokyo, Japan; 2 Department of Clinical Cytology, Kitasato University Graduate School of Medical Sciences, Kanagawa, Japan; 3 Helen Diller Family Comprehensive Cancer Center, University of California San Francisco, San Francisco, California, United States of America; 4 Genome Science Division, Research Center for Advanced Science and Technology, The University of Tokyo, Tokyo, Japan; Cedars-Sinai Medical Center, United States of America

## Abstract

The PI3K (phosphatidylinositol-3-kinase)/mTOR (mammalian target of rapamycin) pathway is frequently activated in endometrial cancer through various PI3K/AKT-activating genetic alterations. We examined the antitumor effect of NVP-BEZ235—a dual PI3K/mTOR inhibitor—and RAD001—an mTOR inhibitor—in 13 endometrial cancer cell lines, all of which possess one or more alterations in *PTEN*, *PIK3CA*, and *K-Ras*. We also combined these compounds with a MAPK pathway inhibitor (PD98059 or UO126) in cell lines with *K-Ras* alterations (mutations or amplification). *PTEN* mutant cell lines without *K-Ras* alterations (n = 9) were more sensitive to both RAD001 and NVP-BEZ235 than were cell lines with *K-Ras* alterations (n = 4). Dose-dependent growth suppression was more drastically induced by NVP-BEZ235 than by RAD001 in the sensitive cell lines. G1 arrest was induced by NVP-BEZ235 in a dose-dependent manner. We observed in vivo antitumor activity of both RAD001 and NVP-BEZ235 in nude mice. The presence of a MEK inhibitor, PD98059 or UO126, sensitized the *K-Ras* mutant cells to NVP-BEZ235. Robust growth suppression by NVP-BEZ235 suggests that a dual PI3K/mTOR inhibitor is a promising therapeutic for endometrial carcinomas. Our data suggest that mutational statuses of *PTEN* and *K-Ras* might be useful predictors of sensitivity to NVP-BEZ235 in certain endometrial carcinomas.

## Introduction

Constitutive activation of the PI3K (phosphatidylinositol 3-kinase) pathway results from various types of alterations, including changes to RTKs (receptor tyrosine kinases), *Ras*, *PIK3CA* (the p110alpha catalytic subunit of PI3K), and *PTEN* [1]. Endometrial cancer is the fourth most frequent cancer in women [2]. There are two pathogenetic types of endometrial carcinomas: estrogen-dependent type I (endometrioid adenocarcinomas) and estrogen-independent type II (high-grade carcinomas). Approximately 80% of endometrial carcinomas are classified as type I [3], [4]. Mutations of *K-Ras* (10–20%), *PTEN* (34–56%), and *PIK3CA* (25–36%) are frequently observed in endometrial cancer [5]–[8]. In addition, we previously revealed that chromosomal imbalances in the Ras-PI3K pathway genes (*NF1*, *PTEN*, *K-Ras*, and *PIK3CA*) are also common in endometrial cancer [9], indicating that the Ras-PI3K pathway is activated in the majority of endometrial cancers.

Novel therapeutics targeting the PI3K/mTOR (mTORC1/2) pathway are being intensively developed. The first clinically approved inhibitors are rapamycin analogs (rapalogs), such as everolimus (RAD001) and temsirolimus, targeting the mTORC1 complex for use with advanced renal cell carcinomas [10]–[13]. However, clinical trials with single-agent rapalog therapies have shown limited response rates in other cancer types [14]. Several potent and selective PI3K inhibitors have recently entered early-phase clinical trials for treatment of various malignant tumors [15]. The limitation of the rapalogs might be explained by the activity of the mTORC1-independent substrates of Akt, including GSK3beta and FOXO1/3a. Rapalogs do not prevent mTORC2-dependent phosphorylation of Akt on Ser-473 or PDK1-dependent phosphorylation of Akt on Thr-308 [16], [17]. In addition, rapalogs may cause feedback activation of the PI3K-Akt pathway mediated by insulin-like growth factor-1 receptor (IGF-1R) signaling [18]. Therefore, a dual PI3K/mTOR inhibition might be a more rational therapeutic option than mTOR inhibition alone in tumors with PI3K-activating mutations.

Developing predictive biomarkers of the PI3K/mTOR inhibitors is important; however, the existence of alterations in the PI3K pathway (or elevated AKT phosphorylation) alone is not necessarily a good biomarker for these compounds. Indeed, tumors with alterations in Ras and RTK do not respond sufficiently to simple PI3K pathway inhibition [19]–[22]. Moreover, multiple genetic alterations in the RTK-Ras-PI3K pathway are reported in many cancers [1]. It remains to be determined which types of alterations are useful as predictive biomarkers.

In this study, we firstly evaluated the antitumor effect of a dual PI3K/mTOR inhibitor, NVP-BEZ235, and an mTOR inhibitor, RAD001 (everolimus), in a panel of endometrial cancer cell lines. Second, we analyzed the antitumor effect of NVP-BEZ235 and RAD001 in vivo. Third, we focused on the predictive biomarkers to the PI3K/mTOR inhibitors, using the mutational status of *K-Ras*, *PTEN*, and *PIK3CA*. Finally, we addressed the antitumor effect of the combined inhibition of the PI3K/mTOR and MAPK pathways in cells with *K-Ras* alterations.

## Materials and Methods

### Cell lines and reagents

Culture conditions of 13 endometrial cancer cell lines (endometrioid adenocarcinomas) were described previously [8]. NVP-BEZ235 and RAD001 (everolimus) were kindly provided by Novartis Pharma AG (Basel, Switzerland). MAPK pathway (MEK) inhibitors PD98059 and UO126 were purchased from Cell Signaling Technology (Beverly, MA).

### PCR and sequencing

The mutational status of 13 cell lines was analyzed by PCR and direct sequencing. The PCR conditions and primers for *PTEN* (exons 1–9), *K-Ras* (exon 1 and 2), and *AKT1* (exon 4) were described previously [8], [23], [24]. The mutational status of *PIK3CA* was analyzed by RT-PCR with LA-Taq according to the manufacturer's protocol (Takara BIO, Madison, WI) to cover entire coding region. The PCR primers were the following: forward, 5′-CCCGAGCGTTTCTGCTTTGGGACAACC-3′; reverse, 5′-AGCGTTTCTGCTTTGGGACAACCATACATC-3′.

### Immunoblotting

Cells were treated with each drug for the indicated time and concentrations and then lysed as described previously [7]. Antibodies to total Akt, phospho-Akt (Ser473), phospho-Akt (Thr308), phospho-GSK3beta (Ser9), total S6, phospho-S6 (Ser235/236, Ser 240/244), p-4EBP1 (Thr37/46), total FoxO1, phospho-FoxO1 (Thr24), phospho-FoxO3a (Thr32), phospho-ERK (ERK1/2-Thr202/Tyr204), total ERK (Cell Signaling Technology, Beverly, MA), and beta-actin (Sigma-Aldrich, St. Louis, MO) were used for immunoblotting, as recommended by the manufacturer, and were detected by an ECL western blot detection kit (Amersham Biosciences, Piscataway, NJ) or Immobilon western detection reagents (Millipore Biosciences, Temecula, CA).

### Proliferation assays

Cell viability assays were performed with the Cell Counting Kit-8 (cell viability colorimetric assay), using the tetrazolium salt WST-8 (2-(2-methoxy-4-nitrophenyl)-3-(4-nitrophenyl)-5-(2,4-disulfophenyl)-2H-tetrazolium, monosodium salt) (Dojindo, Tokyo, Japan), as an MTT (Methyl thiazolyl tetrazorium) assay. In 96-well plates, 1×10^5^ cells were seeded with 10% fetal bovine serum (FBS) and treated with increasing doses of NVP-BEZ235 or RAD001 for 72 h, starting 24 h after seeding. The WST-8 colorimetric assay was quantified at 415 nm and normalized to the value of cells treated with DMSO alone. All experiments were repeated twice.

### Cell cycle analysis

Cells (5×10^5^) were seeded in 60-mm dishes (with 10% FBS) and treated with reagents (such as NVP-BEZ235, RAD001, PD98059, or UO126) for 48 h. Floating and adherent cells were collected by trypsinization and washed twice with PBS. Cells were resuspended in buffer containing ethanol and PBS at a ratio of 7∶3 at −20°C overnight. After being washed twice with PBS, cells were incubated in ribonuclease solution (0.25 mg/mL) (Sigma) for 30 min at 37°C, followed by staining with propidium iodide (50 µg/mL) (Dojindo, Japan) on ice for 30 min in the dark. Cells were then incubated in 70% ethanol at −20°C overnight, treated with 20 µg/mL RNase A, stained with 0.5 µg/mL propidium iodide, and evaluated by flow cytometry (BD FACS Calibur HG, Franklin Lakes, NJ). Cell cycle distribution was analyzed with CELL Quest pro ver. 3.1. (Beckman Coulter Epics XL, Brea, CA). The experiments were repeated 3 times.

### Ethics statement for animal experiments and clinical data

Ethics statement for animal experiments: This study was approved by Animal Care and Use Committee, The University of Tokyo. The approval number is Med-P09-051. Athymic BALB/c mice (CLEA JAPAN, Tokyo, Japan) were maintained in an SPF (Specific Pathogen Free) facility according to our institutional guidelines, and experiments were conducted under an approved animal protocol.

This manuscript includes clinical data, which were previously published elsewhere [7]–[9], [25]. The authors declare that all these participants provided written informed consent, and the study design was approved by the Institutional Review Board of the University of Tokyo Hospital. The approval number is 683.

### Tumor xenografts in nude mice

Subcutaneous xenograft tumors in BALB/c mice were established by the injection of a 500-µL cell suspension of 10×10^6^ AN3CA and HEC-59 endometrial carcinoma cells in PBS. Tumors were removed after exponential growth, cut into 3-mm pieces, and transplanted subcutaneously into other mice. One week after tumor transplantation, mice were assigned randomly to one of three treatment regimens: (1) vehicle (control), (2) NVP-BEZ235, and (3) RAD001. Each treatment group consisted of 6 mice. NVP-BEZ235 and RAD001 were injected orally (p.o.) at daily doses of 40 mg/kg and 2.5 mg/kg, respectively. Tumor volumes (in mm^3^) were calculated by the formula: ([major axis]*[minor axis]2/2). After the treatment, the tumors were removed and analyzed by Western blot analysis. Tumor weight (wet weight) was measured, and the average weight was calculated for each group.

### Single nucleotide polymorphism typing array and array comparative genomic hybridization

A single nucleotide polymorphism (SNP) array was performed in the HEC-6, 50B, 59, 88, 108, 116, 151, and HHUA cell lines. Experimental procedures for GeneChip were performed according to the GeneChip Expression Analysis Technical Manual (Affymetrix, Santa Clara, CA, USA) with the use of a human mapping 250K Nsp array [9]. Array comparative genomic hybridization (CGH) was performed using arrays of 2464 BAC clones (HumArray2.0) in the remaining 5 cell lines (AN3CA, HEC-1B, Ishikawa, KLE, and RL95-2) as described previously [7].

### Statistical analysis

Statistical comparisons of mean final tumor volumes in the xenograft studies were made using a one-way analysis of variance (ANOVA). P<0.05 was considered statistically significant.

## Results

### Classification of endometrial cancer cells according to the mutational status of *PIK3CA*, *PTEN*, and *K-Ras*

**Figure 1:**
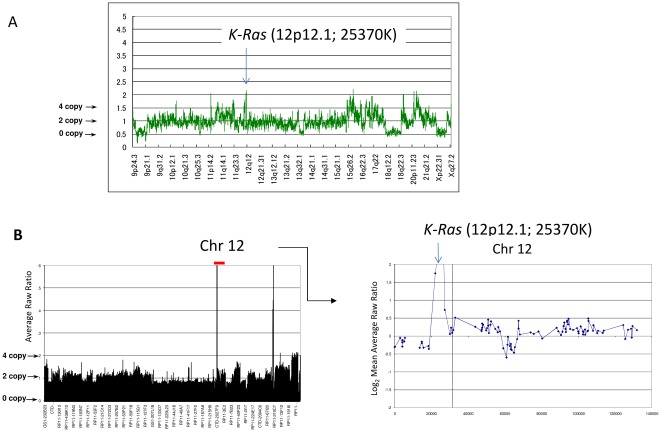
Copy number gain at the locus of ***K-Ras*** **(12p12.1) in the two group D cell lines.** (A) SNP array ‘karyograms’ (250K) of HEC-50B cells. The graph shows the total copy number through chromosome 9p-X. The locus of *K-Ras* is amplified as indicated. (B) Array CGH of KLE cells. The graphs show total copy number throughout the entire genome (Left) and chromosome 12 (Right). The locus of *K-Ras* is amplified as indicated.

We previously reported copy number losses for *PTEN* (26%) and gains for *PIK3CA* (19%) and *K-Ras* (13%) in our 31 clinical samples, in addition to mutations of *K-Ras*, *PTEN*, *PIK3CA*, and *AKT1* [8], [9], [25]. We confirmed that all 13 endometrial cancer cell lines possess one or more alterations (mutations and/or copy number alterations) in the *PIK3CA*, *PTEN*, and *K-Ras* genes (Table 1, Figure 1A and 1B). *AKT1* mutations were not detected in these 13 cell lines. We classified 13 endometrial cancer cell lines into 4 groups according to the mutational status of *PIK3CA*, *PTEN*, and *K-Ras* (Table 1): group A (n = 4), with coexistent mutations of *PIK3CA* and *PTEN*; group B (n = 5), with *PTEN* mutation alone; group C (n = 2), with coexistent mutations of *K-Ras* and *PIK3CA*; and group D (n = 2), with copy number gain of *K-Ras* (without any mutations in these 3 genes). We previously reported that PTEN expression was not detected in *PTEN* mutant endometrial cancer cell lines [8]. We have found no endometrial cell lines without any alterations in the Ras-PI3K pathway, suggesting that this pathway is essentially activated in the majority of endometrial cancer cell lines.

**Table 1:** Classification of endometrial cancer cell lines by mutational status and IC50 values to NVP-BEZ235 and RAD001.

Group	Cell line	Mutational status			Copy number alterations			IC_50_ (nM)	
		*PIK3CA*	*PTEN* (mutated exons)	*K-Ras*	*PIK3CA*	*PTEN*	*K-Ras*	BEZ235	RAD001
A	HEC-116	Mut (R88Q)	Mut 6(M), 7(N)	wild type	nl	nl	nl	19	6
	HEC-6	Mut (R108H)	Mut 4(F), 8(F)	wild type	nl	nl	nl	19	400
	HEC-59	Mut (R38C)	Mut 2(M), 7(N), 7(M), 7(F)	wild type	nl	nl	nl	24	220
	HEC-88	Mut (E365K)	Mut 5(M), 6(M), 8(M), 8(M)	wild type	nl	nl	nl	44	440
B	AN3CA	wild type	Mut 5(N)	wild type	nl	nl	nl	20	14
	Ishikawa	wild type	Mut 8(F), 8(F)	wild type	Gain	nl	nl	30	50
	HEC-151	wild type	Mut 2(M), 4(F)	wild type	nl	nl	nl	51	130
	HEC-108	wild type	Mut 1(F), 8(F)	wild type	nl	nl	nl	55	730
	RL95	wild type	Mut 8(F), 8(F)	wild type	nl	nl	nl	90	>1000
C	HEC-1B	Mut (G1049R)	wild type	Mut (G12D)	Gain	nl	nl	220	200
	HHUA	Mut (R88Q)	Mut 5(F), 8(F)	Mut (G12V)	nl	nl	nl	250	>1000
D	KLE	wild type	wild type	wild type	nl	nl	Gain	110	>1000
	HEC-50B	wild type	wild type	wild type	Gain	nl	Gain	100	>1000

(M) Missense mutation, (N) Non-sense mutation, (F) Frameshift mutation.

### Mutations in *PIK3CA* and/or *PTEN*, in the absence of mutations in *K-Ras*, define the antiproliferative response to NVP-BEZ235 and RAD001 in endometrial cancer cell lines

**Figure 2:**
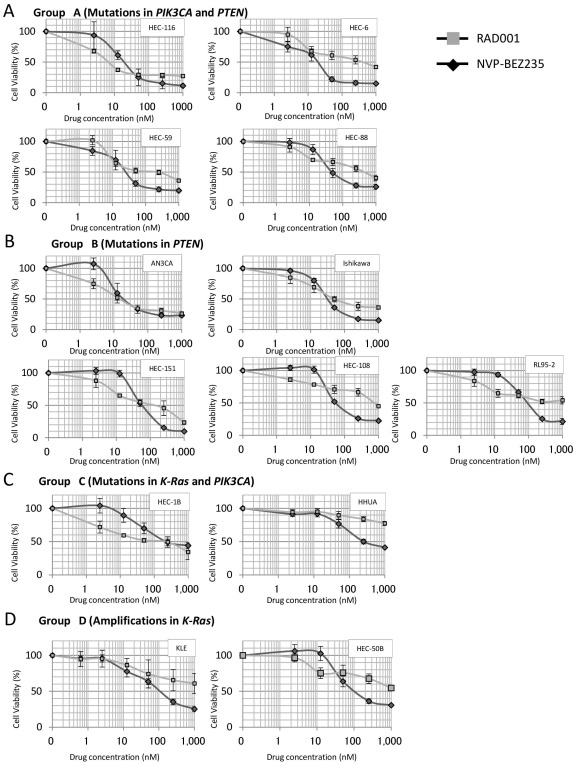
Inhibition of cell proliferation by NVP-BEZ235 and RAD001. (A)–(D) WST-8 assay showing the sensitivity of endometrial cancer cells to NVP-BEZ235 and RAD001 at increasing concentrations of drug (nmol/L) for 72 h. The data was normalized to the value of control cells. (A) Four group A cell lines with double mutations of *PIK3CA* and *PTEN*, (B) Five group B cell lines with *PTEN* mutations, (C) Two group C cell lines with coexistent mutations of *K-Ras* and *PIK3CA*, and (D) Two group D cell lines with chromosomal copy number amplification at the locus of *K-Ras*. All experiments were repeated 3 times, and each value is shown as the mean of 3 experiments ± S.D.

We performed MTT (Methyl thiazolyl tetrazorium) assay by NVP-BEZ235 and RAD001 in the 13 endometrial cell lines. RAD001 (100 nM) showed a growth inhibitory effect against 10 of the 13 cell lines (including all 9 group A and B cell lines), with a 30–70% reduction in cells (Figure 2A–2D). NVP-BEZ235 (100 nM) inhibited cell growth, with a 30–90% reduction in all 13 cell lines. The IC_50_ values for cell proliferation by RAD001 were greater than 100 nM (non-sensitive) in 6 out of the 9 cell lines in groups A and B, whereas the IC_50_ values for NVP-BEZ235 were less than 100 nM (sensitive) in all 9 cell lines in groups A and B (Table 1). Dose-dependent growth suppression was more clearly induced by NVP-BEZ235 than by RAD001 in 8 of the 9 cell lines in groups A and B (Figure 2A–2D). The IC_50_ values for all 4 cell lines in groups C and D were greater than 100 nM for NVP-BEZ235 (Table 1). Taken together, these data show that the existence of *PTEN* and/or *PIK3CA* mutations without *K-Ras* mutations is associated with sensitivity to NVP-BEZ235. In addition, high-dose NVP-BEZ235 might be more broadly effective than RAD001 for treatment of endometrial carcinomas. Growth curves of all cell lines in 1 graph were available for both NVP-BEZ235 and RAD001, respectively (Figures S1 and S2).

### NVP-BEZ235 suppresses phosphorylation of Akt, GSK3beta, S6, and 4EBP1, whereas RAD001 suppresses phosphorylation of S6 and 4EBP1

**Figure 3:**
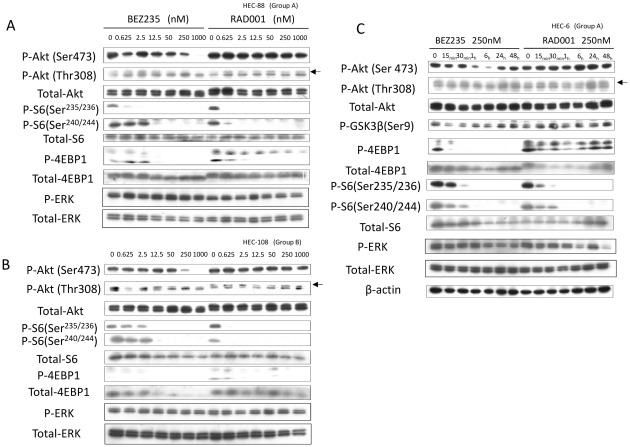
Inhibition of PI3K/mTOR signaling by NVP-BEZ235 and inhibition of mTOR signaling by RAD001 in endometrial cancer cell lines. (A)–(B) Western blot of total lysates of HEC-88 (group A) and HEC-108 (group B) cells, treated with NVP-BEZ235 or RAD001 at concentrations ranging from 0 to 1,000 nmol/L with 10% fetal bovine serum (FBS). phospho-Akt (Ser473, Thr308), phospho-S6 (Ser235/236, Ser240/244), and phospho-4EBP1 (Thr 37/46) levels are shown with total Akt level for a loading control. (C) Western blot of total lysates of HEC-6 (group A) cells treated with NVP-BEZ235 or RAD001 at a dose of 100 nM for up to 48 hours with 10% FBS. Phosphorylation levels of the PI3K/mTOR signaling are shown with loading controls.

We performed immunoblotting with lysates prepared from cells treated with NVP-BEZ235 or RAD001. The phosphorylation (p-) levels of 4E-BP1 and S6 were clearly suppressed by both inhibitors at low concentrations (0.625–2.5 nM). NVP-BEZ235 also suppressed the level of p-Akt (Ser473 and Thr308) (50–1000 nM) in these cells (Figure 3A and 3B). RAD001 did not suppress the phosphorylation level of Akt at any dose (Figure 3A and 3B). The dose dependency of the phosphorylation levels of mTORC1-dependent proteins (4E-BP1 and S6) and Akt suggests that NVP-BEZ mainly works as an mTOR (mTORC1) inhibitor at lower concentrations and functions as a dual PI3K/mTOR inhibitor at higher concentrations.

Next, we performed time-course experiments with NVP-BEZ235 and RAD001. Long-term exposure to NVP-BEZ235 (250 nM) resulted in sustained inhibition of p-S6 and p-4E-BP1. However, the phosphorylation levels of Akt and GSK3beta (an mTORC1-independent protein) recovered nearly to the baseline levels within 24 h (Figure 3C). Exposure to RAD001 resulted in a drastic reduction in the level of p-4EBP1 in 15 min, but the level was recovered within 6 hours; the level of p-S6 was continuously suppressed over the time course (Figure 3C). We confirmed that the phosphorylation level of ERK was not affected by both RAD001 and NVP-BEZ235 (Figure 3A–3C).

### NVP-BEZ235 robustly induces dose-dependent G1 arrest in “sensitive” cells

**Figure 4:**
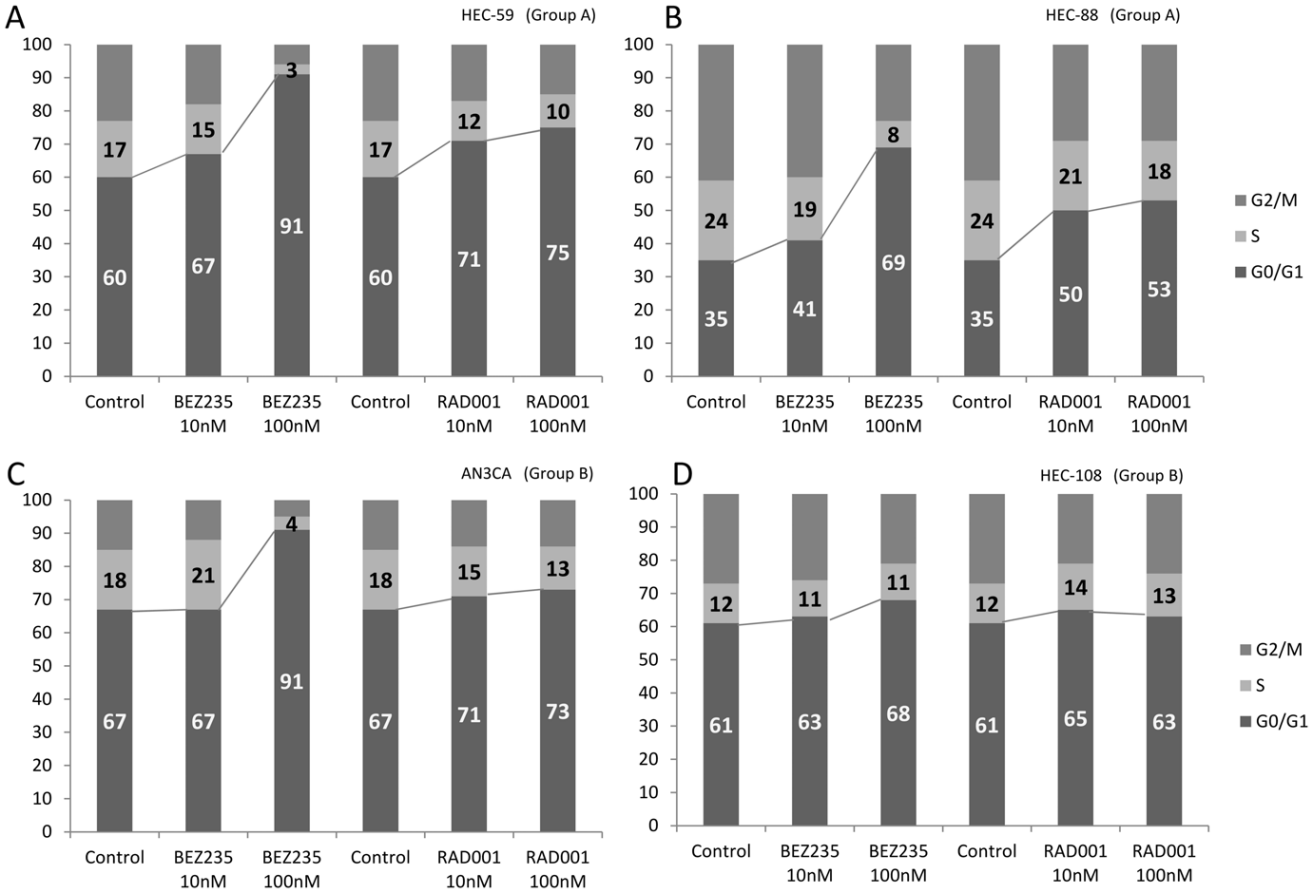
Flowcytometric analysis of cell cycle in cancer cells treated with either NVP-BEZ235 or RAD001. (A–D) Cells (5×10^5^) were seeded in the presence of 10% serum and treated with NVP-BEZ235 or RAD001 for 48 h at a dose of 10 nM or 100 nM, respectively. A higher dose of NVP-BEZ235 (100 nM) significantly augmented the percentage of cells in the G0-G1phase of the cell cycle, compared with that of RAD001 (100 nM). (A)–(B); The data from two group A cells. (C)–(D); the data from two group B cells.

We conducted fluorescence-activated cell sorting (FACS)-based cell cycle analyses before and after NVP-BEZ235 or RAD001 treatment in a subset of the cell lines. At a low concentration (10 nM), G1 arrest was slightly induced by both RAD001 and NVP-BEZ235 (<15%) in group A and group B cell lines (Figure 4A–4D). At a higher concentration (100 nM), G1 arrest was much more effectively induced by NVP-BEZ235 than by RAD001 in three of the four cell lines (Figure 4A–4D). Dose-dependent G1 arrest by NVP-BEZ235 was confirmed in all the other group A and B cell lines (data not shown). G1 arrest was also observed to be induced by either RAD001 or NVP-BEZ235 in the 4 cell lines of groups C and D; however, the dose-dependent effect of NVP-BEZ235 was not significant, except for HEC-1B cells (Figure S3A–S3D). The sub-G1 population was not significantly induced by either inhibitor in all cell lines examined, suggesting that the antitumor effect of these inhibitors is predominantly cytostatic, not cytotoxic.

### In vivo antitumor effect of NVP-BEZ235 and RAD001 in a mouse xenograft model

**Figure 5:**
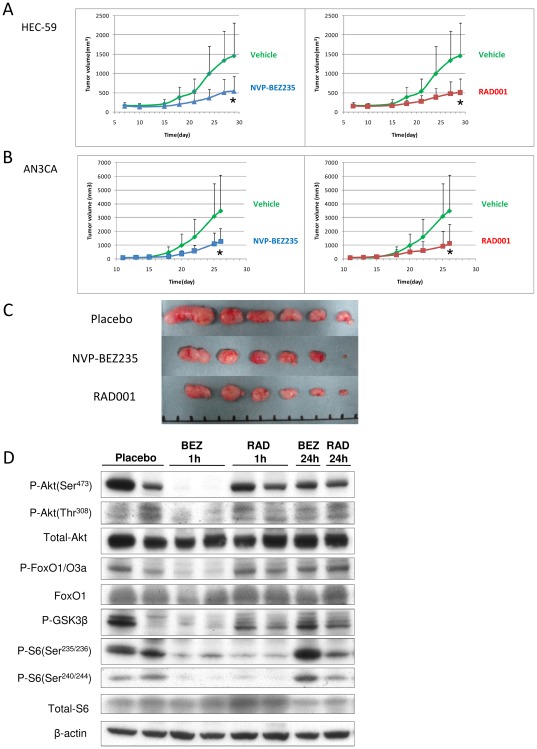
in vivo anti-tumor effect of NVP-BEZ235 and RAD001 in nude mice. Subcutaneous xenograft tumors in athymic BALB/c mice were established in the injection of endometrial carcinoma cells. Mice were treated with a daily dose of 40 mg/kg (NVP-BEZ235) or with a daily dose of 2.5 mg/kg RAD001 or vehicle alone (control). Each treatment group contained 6 mice; (A) HEC-59 and (B) AN3CA. Tumor volumes were calculated by the formula {(major axis)*(minor axis)2/2}mm3 twice a week. Groups were compared at the end of treatment. Points, mean; bars, SD, *;p<0.05. (C) Appearance of subcutaneous tumors in HEC-59 xenografts. (D) Western blot of total lysates from the HEC-59 xenografts. p-Akt, p-FOXO1/3a, p-GSK3beta, p-S6 were assessed 1 and 24 h after the last drug administration. Total Akt and beta-actin were shown as loading controls.

We examined in vivo antitumor activity of both NVP-BEZ235 and RAD001 in mice inoculated with either group A (HEC-59) or group B (AN3CA) cells. Both NVP-BEZ235 and RAD001 significantly suppressed the tumor growth of the xenografts, compared with the control (vehicle). No significant adverse effects, including a body weight loss of more than 10%, were observed in the examined mice (data not shown). Inconsistent with the in vitro data, the effects of NVP-BEZ235 and RAD001 were comparable (Figure 5A–5C).

We then evaluated the phosphorylation levels of the targeted molecules as pharmacodynamic markers. We extracted proteins from the second, third, and fourth largest tumors of each group. Although there were variations in the phosphorylation levels in the control group, NVP-BEZ235 suppressed the phosphorylation levels of Akt, FOXO1/3a, and S6 at 1 h. However, the phosphorylation levels of these proteins recovered to the baseline levels within 24 h (Figure 5D). RAD001 had clearly suppressed the p-S6 level at 1 h, and the effect partly remained at 24 h after the treatment (Figure 5D). Taken together with the in vitro experiments, these results indicate that the antitumor activity of NVP-BEZ235 might not be sufficiently maintained during treatment.

### Inhibition of the MAPK pathway synergistically (or additively) suppresses cell proliferation and induces G1 arrest in *K-Ras* mutant endometrial cell lines

**Figure 6:**
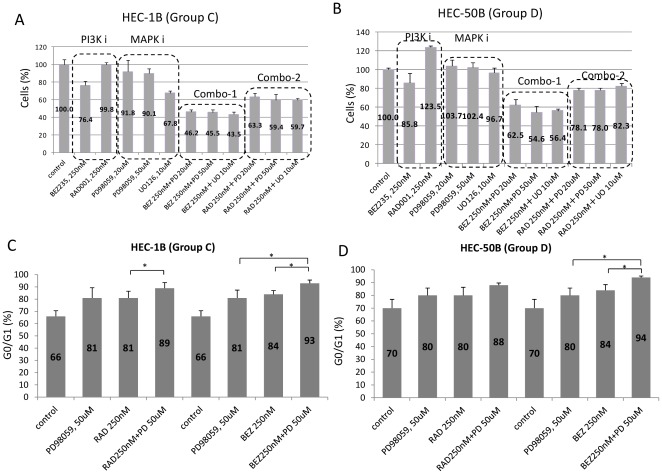
Inhibition of cell proliferation and augmentation of G1 arrest by combination of a MEK inhibitor and NVP-BEZ235 (or RAD001) in cells with alterations in ***K-Ras*** **(mutation or amplification).** (A)–(B) WST-8 assay was performed in HEC-1B (group C) and HEC-50B (group D) cell lines. All the experiments were repeated three times and each value is shown as the mean of three experiments +/− S.D. The combination of a MEK inhibitor (PD98059 or UO126) and NVP-BEZ235 (or RAD001) significantly augmented anti-proliferative effect in these cell lines, compared with either inhibition alone (p<0.05 by Student's t-test). (C)–(D) Flowcytometric analysis of cell cycle in HEC-1B and HEC-50B cells. All experiments were repeated 3 times, and each value is shown as the mean of 3 experiments ± S.D. Combination of a MEK inhibitor (PD98059 or UO126) and NVP-BEZ235 significantly augmented the percentage of cells in the G0-G1phase of the cell cycle in these cells. *: p<0.05 by Student's t-test.

Because the 4 cell lines with *K-Ras* alterations (HEC-1B, HHUA, KLE, and HEC-50B) were less sensitive to both NVP-BEZ235 and RAD001, compared with *K-Ras* wild-type cells with *PTEN* mutations (groups A and B), we combined NVP-BEZ235 (or RAD001) with a MEK inhibitor (PD98059 or UO126) for use in these 4 cell lines (groups C and D). MTT assay revealed that PD98059 (50 µM) or UO126 (10 µM) alone showed a limited growth inhibitory effect with a 10–30% reduction in the 4 cell lines (Figure 6A and 6B, Figure S4A and S4B). However, after treatment with NVP-BEZ235 (250 nM) combined with PD98059 (20–50 µM) or UO126 (10 µM), cell proliferation was suppressed synergistically or additively with a 45–70% reduction (Figure 6A and 6B, Figure S4A and S4B). FACS analysis showed that G1 arrest was markedly induced by a combination of PD98059 (or UO126) and NVP-BEZ235 (Figure 6C and 6D, Figure S4C and S4D). In HEC-1B and HEC-50B cells, the G0/G1 ratio was significantly higher for the combination of PD98059 and NVP-BEZ235 than for either compound alone (Figure 6C and 6D). A similar synergistic effect was also observed with the combination of PD98059 (or UO126) and RAD001 (250 nM), although the effect of RAD001 was weaker than that of NVP-BEZ235 (Figure 6C and 6D, Figure S4C and S4D). The sub-G1 population was not significantly increased in groups C and D cells when using the combination of NVP-BEZ235 and a MEK inhibitor, PD98059 (<5%), as compared to that when using the control or NVP-BEZ235 alone.

### Phosphorylation levels of Akt, S6, and ERK in cells with K-Ras alterations treated with RAD001, NVP-BEZ235, and PD98059

**Figure 7:**
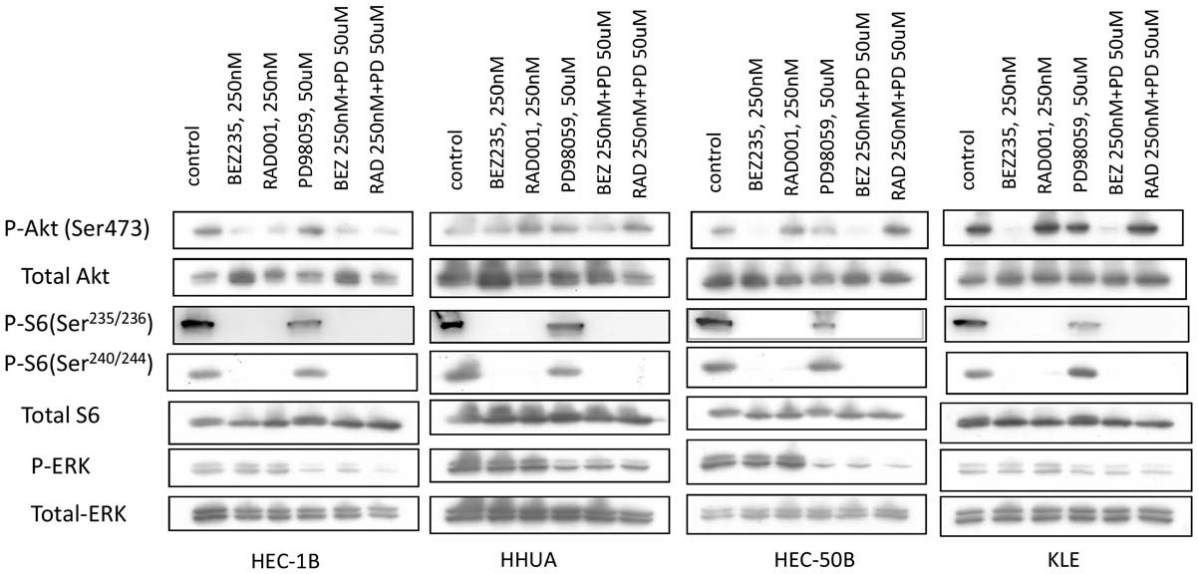
Inhibition of PI3K and MAPK signaling pathway by NVP-BEZ235, RAD001, and a MEK inhibitor (PD98059) in endometrial cancer cell lines with K-Ras alterations. Western blot analysis of total lysates of HEC-1B and HHUA (group C) and HEC-50B and KLE (group D) cells treated with 1 of the PI3K (or MAPK) signal inhibitors or their combination with 10% FBS. Phospho-Akt, phospho-S6, and phospho-ERK levels are shown with total Akt, total S6, and total ERK levels.

We analyzed the PI3K/mTOR and MAPK signaling pathways in cells of groups C and D by western blotting. NVP-BEZ235 at 250 nM suppressed the phosphorylation levels of AKT and S6 in HEC-1B, HEC-50B, and KLE cells. In HHUA cells, the total AKT level was slightly elevated, although the p-AKT level was not significantly changed by NVP-BEZ235 alone (Figure 7). RAD001 at 250 nM suppressed the p-S6 level in all cells of groups C and D (Figure 7). Both RAD001 and NVP-BEZ235 did not suppress the p-ERK level, whereas a MEK inhibitor, PD98059, decreased the p-ERK level in these cells.

## Discussion

We examined activity of the PI3K/mTOR pathway inhibitors in endometrial cancer cell lines with a particular focus on (i) the antitumor effect of an mTOR inhibitor (RAD001) and a dual PI3K/mTOR inhibitor (NVP-BEZ235), (ii) predictive biomarkers of the mutational status of the PI3K pathway genes, and (iii) combined inhibition of the MAPK pathway and the PI3K/mTOR pathway in *K-Ras* mutant cells. MTT assay and FACS analysis in a panel of endometrial cancer cell lines revealed a clear dose-dependent effect of NVP-BEZ235 on cell proliferation. NVP-BEZ235 induces G1 arrest much more efficiently at a higher concentration (100 nM) than at a lower concentration (10 nM). In contrast, RAD001 does not show evidence of such dose dependency. Previous reports also suggested that NVP-BEZ235 was more effective than rapalogs at higher concentrations [26], [27]. PI3K activity might not be sufficiently suppressed by 100 nM NVP-BEZ235, as indicated by the observation that decreased phosphorylation of Akt (Thr308) is not observed at 50 nM but is observed at 250 nM or higher. In addition, IC_50_ values were under 100 nM in cells from groups A and B. These data are in agreement with previous reports on other cancers that indicate a discrepancy between the basal activity of the PI3K/Akt pathway and the biochemical activity of NVP-BEZ235 [26]–[29]. Nevertheless, the dose-dependent antiproliferative activity at concentrations ≥250 nM suggests that the effect of NVP-BEZ235 was, at least in part, caused by inhibition of the PI3K/Akt pathway. Our data suggest that a dual inhibitor of PI3K/mTOR might be a more promising therapeutic strategy than a single mTOR inhibitor in endometrial cancer.

Our in vivo studies in 2 cell lines of xenograft mice support the in vitro findings that inhibition of the PI3K/mTOR axis has an antitumor effect in endometrial cancers. We did not see any superior efficacy of NVP-BEZ235 in the in vivo study. The concentrations we used were 40 mg/kg for NVP-BEZ235 and 5 mg/kg for RAD001, which are equivalent with the previous in-vivo experiments [26], [28], [30]–[32]. In a pharmacodynamic analysis, the levels of p-Akt, p-GSK3beta, p-FOXO1/3a, and p-S6 in tumors returned to the baseline levels within 24 h after administration of NVP-BEZ235, suggesting that inhibition of PI3K signaling by NVP-BEZ235 might not be sufficiently maintained over time. This is compatible with previous data showing that inhibition of p-Akt (Ser473) was maintained for 16 h, with recovery to baseline levels at 24 h [33]. It remains to be determined which oral dosing schedule is optimal in treatment of endometrial cancer. As well, the mechanisms of in-vivo anti-tumor effect by these drugs should be more clarified, as inhibition of mTOR might result in anti-angiogenic effect by suppressing HIF1-VEGF pathway [34].

Developing predictive biomarkers in therapeutics targeting the PI3K/mTOR pathway is crucial, as alterations in several molecules are involved in the activation of this pathway. *PIK3CA* mutation and HER2 amplification have been recommended as useful biomarkers in breast cancer [26], [35], [36]. Mutant oncogenic Ras has been suggested as a dominant determinant of resistance in several solid tumor cells [19], [37]. PTEN deficiency is controversial as a predictive biomarker [26], [36], [38]. The mechanism of resistance in PTEN-deficient tumors might be explained by the predominant activation of p110beta in *PTEN* mutant tumors [39], [40], as NVP-BEZ235 and most of the other PI3K inhibitors suppress p110beta less preferentially than the other p110 isoforms. However, p110beta is not a predominant isoform in endometrial carcinomas with *PTEN* mutations [8]. The significance of p110alpha in *PTEN* mutant endometrial cancer would be helpful to identify patients susceptible to NVP-BEZ235. Thus, the existence of *PTEN* mutations might be a predictive biomarker for the PI3K/mTOR inhibitors in endometrial carcinomas. Further in vivo analysis, including the anti-tumor effect of NVP-BEA235, RAD001, or a combination of these compounds with a MEK inhibitor on groups C and D tumors would be necessary to evaluate the utility of these factors as biomarkers.

Feasibility of mutational analysis of the predictor genes should be also considered in clinical trials. *K-Ras* mutational analysis would be reasonably achievable, as “hot spot” mutations are located only in 2 exons (codons 12, 13, and 61). However, mutations of *PIK3CA* and *PTEN* are widespread in the entire coding region. Others and we have reported that PTEN expression is sufficiently evaluated by immunohistochemistry and is correlated with mutational status [25], [41]. A combination of screening *K-Ras* mutations and immunohistochemistry analysis of PTEN might be a useful and feasible strategy in clinical trials of endometrial cancer. We previously reported that *PIK3CA* mutations frequently coexist with *K-Ras* muations in endometrial cancer [8]. The two group C cells (HEC-1B and HHUA) with double mutations of *PIK3CA* and *K-Ras* were less sensitive to NVP-BEZ235, compared with group A/B cells. Thus, *PIK3CA* mutation alone might not be a good biomarker in endometrial cancer. Over 5 clinical studies of the rapalogs have been developed in advanced endometrial cancer. Of them, Oza et al reported phase II study of temsirolimus in endometrial cancer, showing clinical benefit rate (partial response and stable disease) of 52% in chemoetherapy-treated patients [42]. They suggested that PTEN loss (immunohistochemistry and mutational analysis) and molecular markers of PI3K/Akt/mTOR pathway did not correlate with the clinical outcome. It would be clarified whether information of *K-Ras* alterations might be helpful in the clinical settings. As well, further exploration of the other PI3K-activating alterations (such as mutations of *FGFR2* and *PIK3R1*) and other measurable characteristics (such as standardized uptake value by PET imaging) would be also necessary to establish the most useful clinical biomarkers.

She et al reported that various cell lines with double mutations of *K-Ras*/*BRAF* and *PIK3CA* were resistant to AKT inhibitors, and suggested that a MEK inhibitor sensitizes these double mutant cells to AKT or PI3K inhibitors [43]. Our data in the two group C cells support that a combination of a MEK inhibitor and a PI3K (AKT) inhibitor might be effective to various types of cancers with double mutations of *K-Ras* and *PIK3CA*. In addition to mutations, copy number gain of oncogenes is also important for “oncogene addiction”. We previously reported that extensive chromosomal instability (with 5 or more copy number alterations) is a poor independent prognostic factor in endometrial carcinomas [10]. Although extensive chromosomal instability is more common in type II endometrial carcinomas [44], the percentage of extensive chromosomal instability was 31% in our clinical endometrioid adenocarcinoma samples [10]. We found that both group D cell lines (KLE and HEC-50B) harbor extensive CNAs (copy number alterations), with copy number gain at the locus of *K-Ras*, although they do not possess any mutations in *K-Ras*, *PTEN* and *PIK3CA*. The antiproliferative effect of combined inhibition of MAPK pathway and PI3K/mTOR pathway in group D cells suggests that this combination therapy might be an option to treat tumors with CNA in *K-Ras*. The dual inhibition of the PI3K and MAPK pathways might overcome the resistance to PI3K/mTOR inhibition alone in certain endometrial tumors with *K-Ras* alterations through its enhanced cytostatic effect (at least in part). Cheung et al reported that endometrial cell lines with wild-type PI3K pathway members were resistant to an mTOR inhibitor, rapamycin, suggesting that other unexamined factors, including CNA in *K-Ras*, might be involved in the anti-tumor effect of rapalogs [45]. Phosphorylation of 4E-BP1 is not only regulated by mTORC1, but also by ERK signaling [43], [46], suggesting the crosstalk between PI3K/mTOR pathway and MAPK pathway. It would be necessary to evaluate the in vivo effect of the combined therapy in tumors with *K-Ras* alterations to address the activity of the MAPK pathway in endometrial cancer.

## Supporting Information

Figure S1 **Inhibition of cell proliferation by NVP-BEZ235 in 13 endometrial cancer cells.** The growth curves of all the 13 cells in response to NVP-BEZ235 in the WST-8 assay (in Figure 2) are shown in one graph.
(PPT)

Figure S2 **Inhibition of cell proliferation by RAD001 in 13 endometrial cancer cells.** The growth curves of all the 13 cells in response to RAD001 in the WST-8 assay (in Figure 2) are shown in one graph.
(PPT)

Figure S3 **Flowcytometric analysis of cell cycle in cancer cells treated with either NVP-BEZ235 or RAD001.** (A–D) Cells (5×10^5^) were seeded and treated with NVP-BEZ235 or RAD001 for 48 h at a dose of 10 nM or 100 nM, respectively, as described in Figure 4. (A)–(B); The data from the two group C cells (HEC-1B and HHUA). (C)–(D); the data from the two group D cells (KLE and HEC-50B).
(PPT)

Figure S4 **Inhibition of cell proliferation and augmentation of G1 arrest by combination of a MEK inhibitor and NVP-BEZ235 (or RAD001) in cells with alterations in** ***K-Ras*** **(mutation or amplification).** (A)–(B) WST-8 assay was performed in HHUA (group C) and KLE (group D) cell lines. (C)–(D) Flowcytometric analysis of cell cycle in HHUA (group C) and KLE (group D) cells. All experiments were repeated 3 times, and each value is shown as the mean of 3 experiments ± S.D.
(PPT)
