# Supplementary figures and images for: Genotype-Dependent Efficacy of a Dual PI3K/mTOR Inhibitor, NVP-BEZ235, and an mTOR Inhibitor, RAD001, in Endometrial Carcinomas
Source: PLoS One. 2012 May 25;7(5):e37431. doi: 10.1371/journal.pone.0037431 (PMC3360787; doi:10.1371/journal.pone.0037431)

## Slide 1
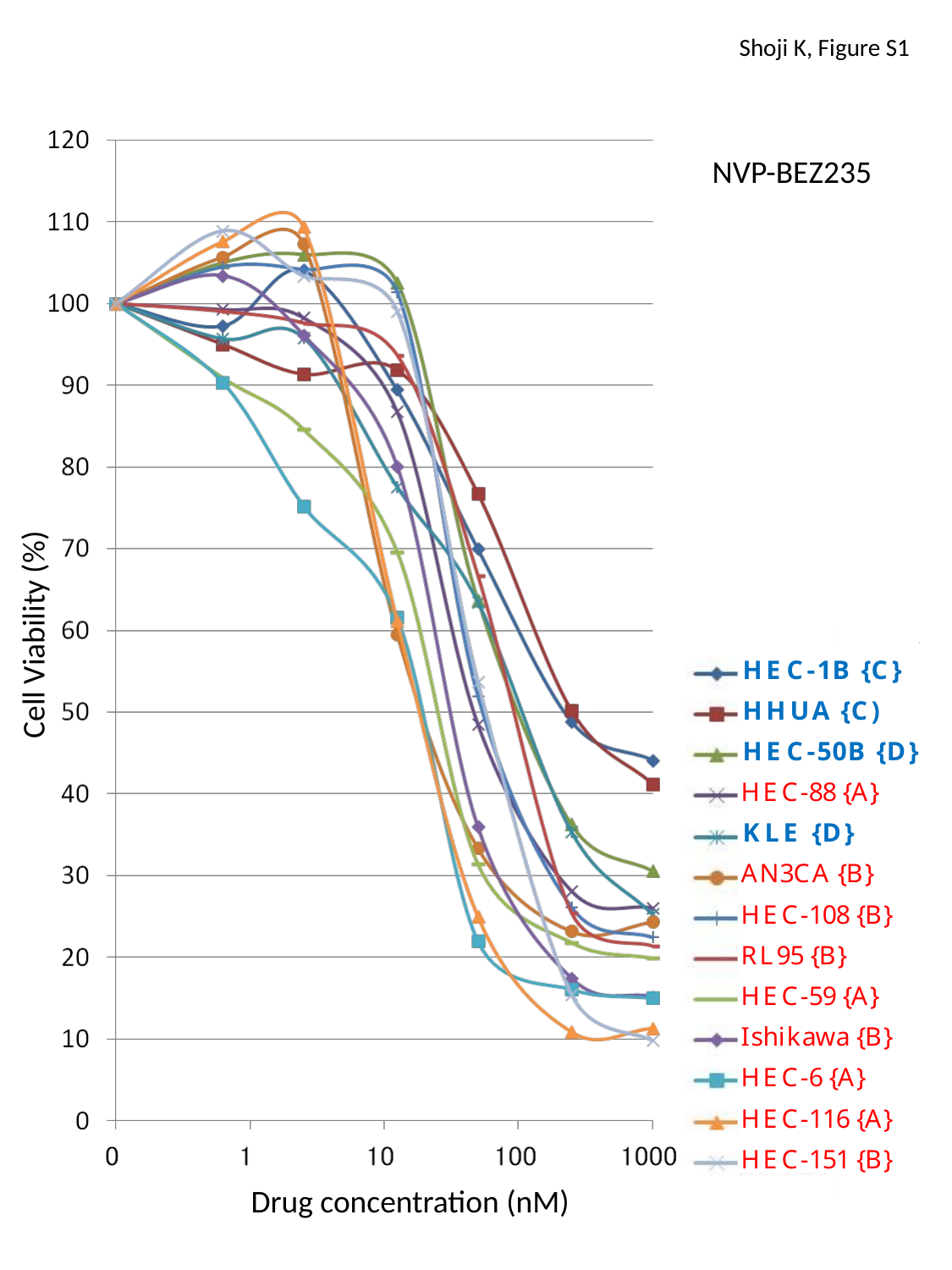

Shoji K, Figure S1
NVP-BEZ235
Cell Viability (%)
Drug concentration (nM)

Supplement: Figure S1 — Inhibition of cell proliferation by NVP-BEZ235 in 13 endometrial cancer cells. The growth curves of all the 13 cells in response to NVP-BEZ235 in the WST-8 assay (in Figure 2) are shown in one graph. (PPT) [file pone.0037431.s001.ppt]

## Slide 1
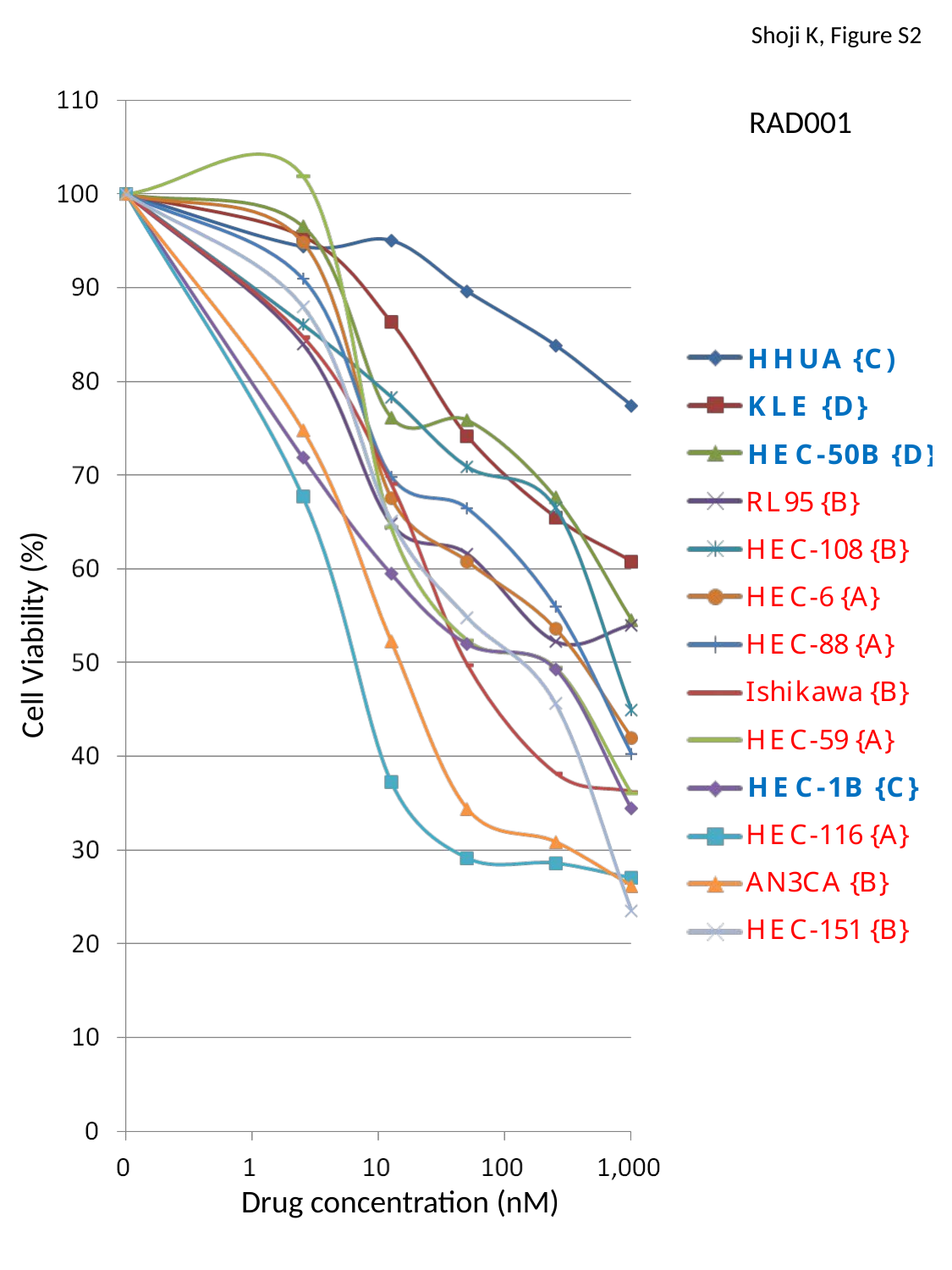

Shoji K, Figure S2
RAD001
Cell Viability (%)
Drug concentration (nM)

Supplement: Figure S2 — Inhibition of cell proliferation by RAD001 in 13 endometrial cancer cells. The growth curves of all the 13 cells in response to RAD001 in the WST-8 assay (in Figure 2) are shown in one graph. (PPT) [file pone.0037431.s002.ppt]

## Slide 1
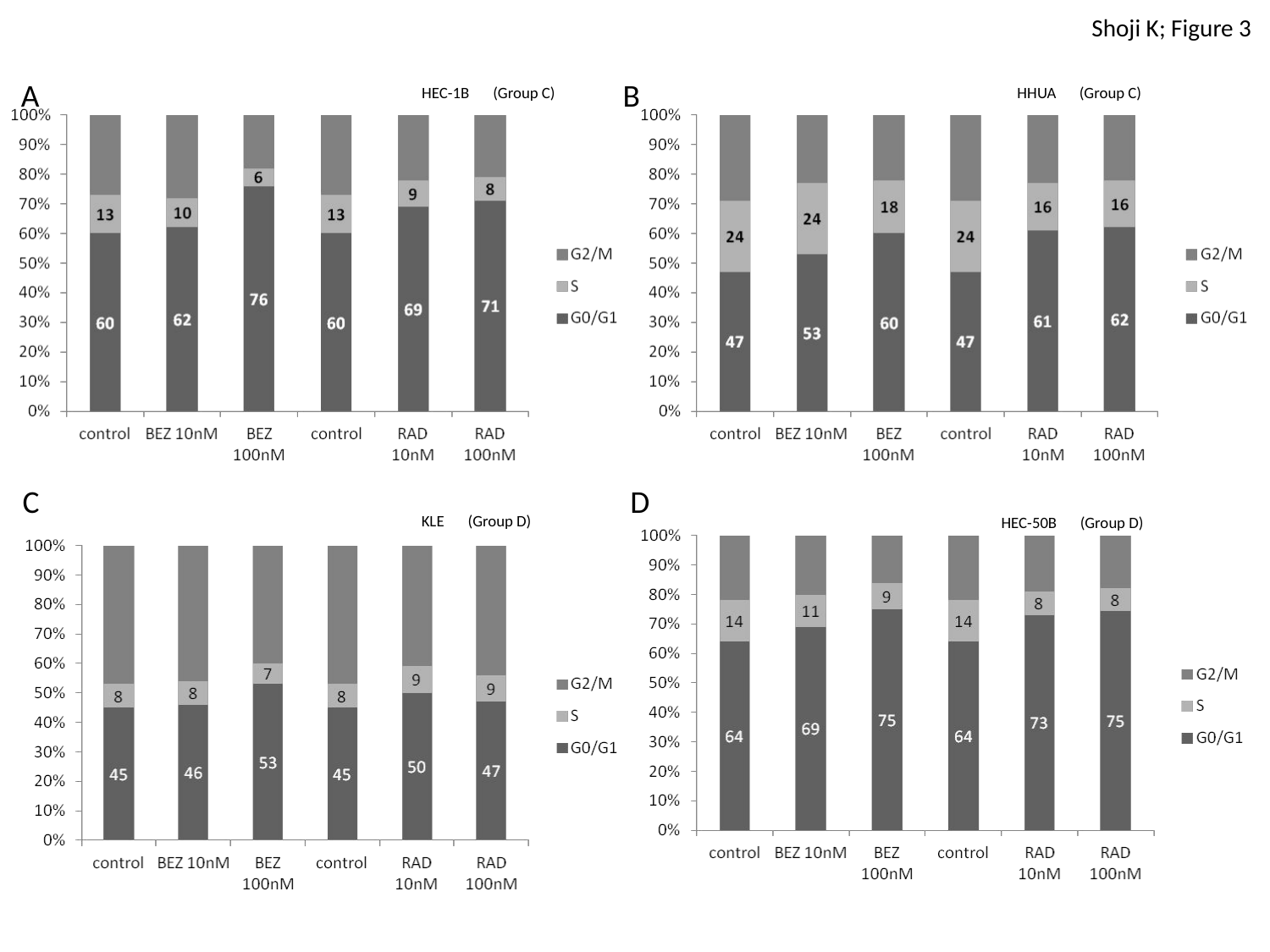

Shoji K; Figure 3
A
B
HEC-1B　(Group C)
HHUA　(Group C)
C
D
KLE　(Group D)
HEC-50B　(Group D)

Supplement: Figure S3 — Flowcytometric analysis of cell cycle in cancer cells treated with either NVP-BEZ235 or RAD001. (A–D) Cells (5×10^5^) were seeded and treated with NVP-BEZ235 or RAD001 for 48 h at a dose of 10 nM or 100 nM, respectively, as described in Figure 4. (A)–(B); The data from the two group C cells (HEC-1B and HHUA). (C)–(D); the data from the two group D cells (KLE and HEC-50B). (PPT) [file pone.0037431.s003.ppt]
