# Supplementary material for: Genotype-Dependent Efficacy of a Dual PI3K/mTOR Inhibitor, NVP-BEZ235, and an mTOR Inhibitor, RAD001, in Endometrial Carcinomas
Source: PLoS One. 2012 May 25;7(5):e37431. doi: 10.1371/journal.pone.0037431 (PMC3360787; doi:10.1371/journal.pone.0037431)
Supplement: Figure S4 — Inhibition of cell proliferation and augmentation of G1 arrest by combination of a MEK inhibitor and NVP-BEZ235 (or RAD001) in cells with alterations in K-Ras (mutation or amplification). (A)–(B) WST-8 assay was performed in HHUA (group C) and KLE (group D) cell lines. (C)–(D) Flowcytometric analysis of cell cycle in HHUA (group C) and KLE (group D) cells. All experiments were repeated 3 times, and each value is shown as the mean of 3 experiments ± S.D. (PPT) [file pone.0037431.s004.ppt]

## Slide 1
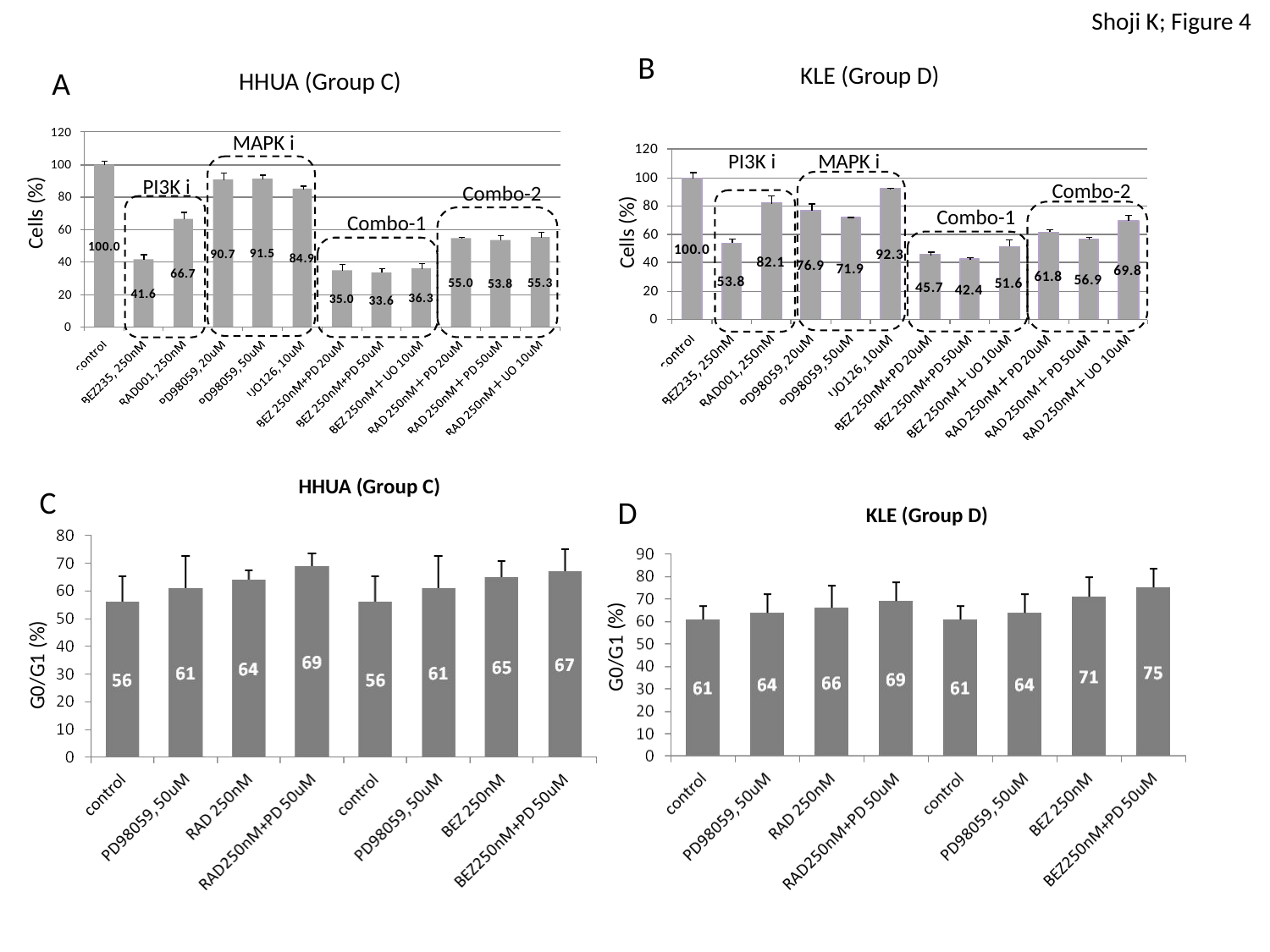

Shoji K; Figure 4
B
KLE (Group D)
A
HHUA (Group C)
MAPK i
PI3K i
MAPK i
PI3K i
Combo-2
Combo-2
Cells (%)
Combo-1
Combo-1
Cells (%)
HHUA (Group C)
C
D
KLE (Group D)
G0/G1 (%)
G0/G1 (%)
